# Communication interventions to promote the public’s awareness of antibiotics: a systematic review

**DOI:** 10.1186/s12889-019-7258-3

**Published:** 2019-07-08

**Authors:** Valerie R. Burstein, Renee P. Trajano, Richard L. Kravitz, Robert A. Bell, Darshan Vora, Larissa S. May

**Affiliations:** 1 Ohio State University College of Medicine, Columbus, OH USA; 2 University of California Davis, Davis, CA USA; 3 University of California Davis, Sacramento, CA USA; 4 The George Washington University School of Medicine & Health Sciences, Washington, DC, USA

**Keywords:** Antibiotics, Messaging programs, Public awareness

## Abstract

**Background:**

Inappropriate antibiotic use is implicated in antibiotic resistance and resultant morbidity and mortality. Overuse is particularly prevalent for outpatient respiratory infections, and perceived patient expectations likely contribute. Thus, various educational programs have been implemented to educate the public.

**Methods:**

We systematically identified public-directed interventions to promote antibiotic awareness in the United States. PubMed, Google Scholar, Embase, CINAHL, and Scopus were queried for articles published from January 1996 through January 2016. Two investigators independently assessed titles and abstracts of retrieved articles for subsequent full-text review. References of selected articles and three review articles were likewise screened for inclusion. Identified educational interventions were coded for target audience, content, distribution site, communication method, and major outcomes.

**Results:**

Our search yielded 1,106 articles; 34 met inclusion criteria. Due to overlap in interventions studied, 29 distinct educational interventions were identified. Messages were primarily delivered in outpatient clinics (*N* = 24, 83%) and community sites (*N* = 12, 41%). The majority included clinician education. Antibiotic prescription rates were assessed for 22 interventions (76%). Patient knowledge, attitudes, and beliefs (KAB) were assessed for 10 interventions (34%). Similar rates of success between antibiotic prescription rates and patient KAB were reported (73 and 70%, respectively). Patient interventions that did not include clinician education were successful to increase KAB but were not shown to decrease antibiotic prescribing. Three interventions targeted reductions in *Streptococcus pneumoniae* resistance; none were successful.

**Conclusions:**

Messaging programs varied in their designs, and many were multifaceted in their approach. These interventions can change patient perspectives regarding antibiotic use, though it is unclear if clinician education is also necessary to reduce antibiotic prescribing. Further investigations are needed to determine the relative influence of interventions focusing on patients and physicians and to determine whether these changes can influence rates of antibiotic resistance long-term.

**Electronic supplementary material:**

The online version of this article (10.1186/s12889-019-7258-3) contains supplementary material, which is available to authorized users.

## Background

Antibiotic resistance is a worldwide threat fueled in large part by the inappropriate use of antibiotics. According to the Centers of Disease Control and Prevention (CDC), over 2 million people are affected by antibiotic-resistant infections in the United States each year. [1] Annually, at least 23,000 people die as a direct result of these infections. [1] The overuse of antibiotics is particularly prevalent in outpatient settings. Antibiotics are prescribed in approximately 10% of all ambulatory visits, most commonly for respiratory conditions. [2] Although justified in some cases, antibiotics are overprescribed for respiratory infections, which are mostly viral in nature. A major proportion of these prescriptions are inappropriate, [3–5] and broad spectrum antibiotics are more likely to be prescribed than more narrowly targeted alternatives. [2] Patient expectation has been thought to play a significant role in the excess use of antibiotics. Although information and reassurance may be as important to patients as receipt of antibiotics, [6] perceived expectations do alter physician behavior. [7, 8] Most physicians feel pressured to prescribe when there is a patient expectation for antibiotics. [9] One study demonstrated that patients expecting antibiotics were three times more likely to receive them, and physicians were ten times more likely to prescribe if they perceived that the patient expected antibiotics. [7] Further, physicians often perceive these expectations in the absence of verbal requests. [10]

To reduce antibiotic resistance and adverse effects for patients, it is essential to reduce inappropriate antibiotic use. The evolution of antibiotic resistance is multifactorial, and there is no single intervention that can solve this public health threat. However, evidence suggests that antibiotic stewardship programs can curtail excessive antibiotic use, reduce antibiotic-associated events, decrease antibiotic resistance, and improve patient outcomes. [11, 12] Implementation of such programs requires education of the public and partnership between patients and providers due to concerns regarding patient satisfaction and the patient-provider relationship. [13]

Various educational interventions have been implemented with the goal of increasing antibiotic awareness. The objective of this study was to systematically identify, characterize, and evaluate the messaging approaches used in these interventions. We were interested in interventions in the United States, specifically, for a variety of reasons. For one, antibiotic use and resistance rates varies widely between countries. [14] Further, the United States lacks a unified health care system, potentially adding barriers to the implementation of public health interventions. We included studies investigating interventions directed at patients or the general public to analyze their relevance in United States outpatient settings. Future efforts to curtail inappropriate antibiotic use and ultimately reduce antibiotic resistance can be improved by identifying the features of successful interventions.

## Methods

We included articles investigating a public campaign or patient-directed messaging program to promote awareness of appropriate antibiotic use within the United States.

### Search strategy

We developed independent literature search strategies for Pubmed, Google Scholar, Embase, CINAHL, and Scopus. These search protocols utilized combinations of relevant keywords or phrases appropriate for each database regarding antibiotics, messaging, and public awareness to retrieve articles published from January of 1996 through January of 2016 (Additional file 1: Appendix A). Relevant search terms were identified using Pubmed Medical Subject Headings (MeSH).

### Selection

After initial retrieval, article abstracts were independently coded by two investigators for eligibility based on predefined inclusion criteria. Discordance was resolved by a third investigator. To be included, the interventions must have been conducted within the United States and had a patient or public education aimed at enhancing antibiotic awareness, including indications, risks, and/or importance of usage according to instruction. Additionally, the study must have formally evaluated the program in terms of knowledge, attitudes, and/or beliefs; adherence to recommended treatment; resistance patterns; or prescribing practices. Retrieved articles were assessed for inclusion based on title and abstract, followed by full-text review.

**Fig. 1:**
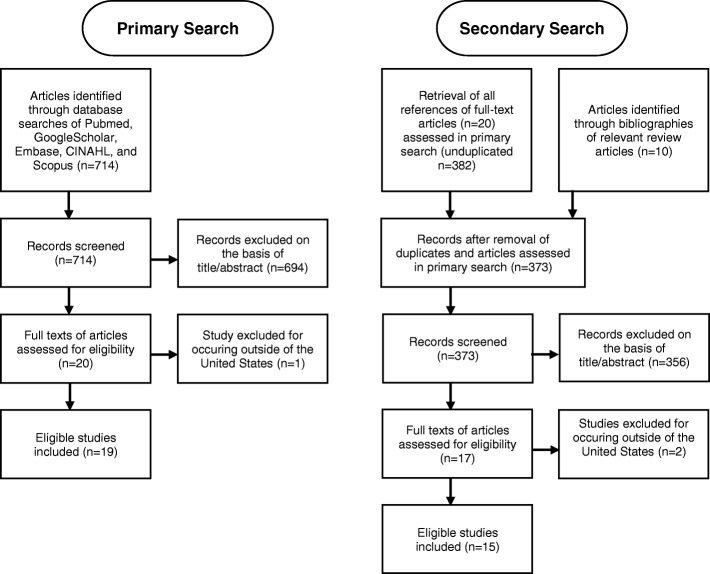
Application of primary and secondary search strategies to retrieve total number of studies for analysis

To locate studies that may have been missed using the search strategies, a secondary search was performed. We retrieved all the references of the articles found in the primary search. Three relevant review articles were also inspected for educational interventions within the United States. [15–17] Eligibility for inclusion was assessed by two independent investigators on the basis of title and abstract, followed by full text review (Fig. 1).

At the end of this process, 34 articles were included in the analysis. [18–51]

### Coding of intervention features

Selected articles were first classified according to study design (Additional file 1: Appendix B). Randomized controlled trials included both individual and group (cluster) randomization. Next, educational interventions were identified from all 34 articles. The articles overlapped in the interventions that they analyzed. The 34 articles evaluated 29 antibiotic communication interventions, as different outcomes for 4 of the interventions were analyzed in separate articles (Additional file 1: Appendix C).

The 29 interventions were codified by the targeted audience, content of the messages, form of communication, and primary outcomes. Two investigators coded the interventions’ features. A third investigator coded interventions for which there were any discrepancies.

#### Target audience

Each intervention was coded for whether it targeted adults generally, parents, children, and/or Spanish-speaking persons.

#### Message content

Content was assessed by determining if the provided information: 1) covered appropriate use of antibiotics; 2) covered risk of inappropriate use; or 3) relied on CDC materials or principles. “Appropriate use” was defined as either education about differences between viruses and bacteria or the indications for antibiotics. “Risks of inappropriate use” was defined as information regarding possible antibiotic adverse events or antibiotic resistance. Reliance on CDC included the stated use of CDC principles or CDC employees in the development of materials or the direct use of CDC materials.

#### Message distribution

Educational interventions were assessed for the following location(s) of message distribution: emergency department, outpatient clinical setting (office, clinic, urgent care), community setting, and/or personal residence. “Community settings” was defined to include childcare centers, local pharmacies, community fairs, restaurants, churches, and schools. Messages distributed by public media, such as television or radio, were classified under “community site.” Correspondence mailed directly to specific households was classified separately.

#### Form of communication

Educational messages were analyzed for their mode of communication, including the use of print media (posters, handouts), media directly mailed to individuals, public media (radio, television, newspaper ads or stories, outdoor advertising), video messaging, Short Message Service (SMS) messaging, and/or presentations.

#### Outcomes

All outcomes targeted were coded within these categories: patient or parent knowledge, attitude, and/or beliefs; physician knowledge, attitude, and/or beliefs; antibiotic prescribing; appropriateness of antibiotic prescriptions; adherence to recommended treatment; drug resistance patterns; drug costs and/or healthcare utilization. Outcome effectiveness was reported as defined by the original articles, and the percent effective was determined for both the total number of studies as well as for randomized-controlled trials alone.

#### Clinician education

If an intervention included messages for the medical community, these messages were also characterized. We coded for the presence or absence of messages targeting physicians, nurses, physician assistants, and nurse practitioners. Messaging content was described as risks of inappropriate use; pathogen resistance surveillance data; algorithms, pathways, and/or guidelines; audit and feedback; making use of CDC principles; and/or “cold kit” distribution (supplies for symptomatic treatments of viral illnesses). The location of distribution was similarly described. Modes of message distribution were analyzed for the use of computer-decision support, group sessions or presentations, email, mailings, and/or continuing medical education (CME).

### Statistical analysis

Intervention characteristics were analyzed using descriptive statistics.

### Reliability of coding

#### Primary search

Interrater reliability for inclusion/exclusion of candidate articles based on title and abstract using Cohen’s Kappa was high (κ = 0.74 95% CI, 0.63 to 0.85); cases of discordance were resolved by a third investigator. A full-text review was then performed for final selection (κ = 1.0).

#### Secondary search

Articles localized via our secondary search strategy were likewise assessed for further review based on title and abstract (κ = 0.65 95% CI 0.45 to 0.85), and discordance was resolved by a third investigator. Reviewers independently performed a full-text review of these articles for final selection (κ = 1.0).

## Results

### Article study design

Fourteen of the 34 included articles (41%) were randomized controlled trials, the gold standard for intervention effects. Another 18 articles were prospective studies (53%). Of these non-randomized trials, 14 included a comparison population to distinguish the intervention-attributable effect from secular trends, while 4 studies simply compared post- and pre-intervention measurements. One study used multilevel logistical regression of intercept interview data to analyze changes in knowledge associated with the intervention. Lastly, one article used reports from 3 separate organizations to describe changes in antibiotic use, but no statistics were provided to indicate the significance of these results.

### Total educational interventions

The 29 educational interventions were stratified based on primary audience, content of messages, location of distribution, mode of communication, and outcomes studied (Additional file 1: Appendix D).

#### Target audience

All 29 interventions aimed to educate adults, and the majority (62%) specified parents as the primary target. In total, 31% of studies included materials in the Spanish language; only one exclusively targeted Latinos residing the United States. [40]

#### Content of messages

Not all articles described the content of their materials. However, the majority of interventions (83%) explicitly specified educational messages regarding the appropriate use of antibiotics, such as the difference between viruses and bacteria and the specific illnesses for which antibiotics are indicated. Risks of inappropriate use, such as antibiotic resistance and potential side effects, were also commonly communicated (72%). Most interventions noted the use of Center for Disease Control (CDC) principles or materials (69%).

#### Setting/mode of distribution

Educational messages were distributed in a variety of settings. On average, messages were distributed in 1.6 coded locations per intervention (emergency department, office/clinic/urgent care, community site, and/or personal residence). The majority of interventions distributed some or all their content in an office or clinic setting (83%). A substantial proportion (41%) of the interventions distributed messages within the community (community pharmacies, child care centers, schools, etc.). Interventions used on average 1.9 methods of communication (coded methods included print media, mailed media, public media, video message, SMS message, and presentations). About a quarter of interventions used public media to reach their audience. However, handouts and posters were the most commonly used mode of distribution (90% of interventions).

#### Outcomes

Interventions were varied in their desired outcomes, and many had several major outcomes. Overall, 76% (22/29) of interventions observed favorable changes for at least one of the major outcomes studied.

Antibiotic prescription rates were most commonly studied (22 of 29 interventions). This was often measured by prescriptions per visit, prescriptions per visit for respiratory illnesses, prescriptions per physician, or prescriptions per person of community or health organization. Of these interventions, 73% reported reductions in antibiotic prescribing. However, several studies observed significant changes in antibiotic prescribing that were attributable to secular trends. Thus, Tables 1 and 2 summarize the trials that demonstrated intervention-attributable prescription rate declines compared to a control. Table 1 includes randomized controlled trials, the gold standard of study designs. Randomized controlled trials comprised only 7 of the 22 studies that analyzed antibiotic prescribing (32%). Among randomized controlled trials, 71% resulted in decreased antibiotic prescribing compared to the control (5 of 7 interventions). One of these studies [41] simply showed less of an increase in antibiotic use compared to the control group, however, and is thus not included in Table 1. Table 2 includes quasi experimental studies that were successful compared to a control population.

**Table 1:** Randomized Controlled Trials Demonstrating Decreased Antibiotic Prescription Rates in Response to Patient- or Public- Centered Educational Interventions

Location of Intervention (years observed)	Setting	Patient Education	Provider Education	Outcome Measure	Prescription Rate Change			
					Control	(Full) Intervention	Intervention Effect	*P* value (intervention effect compared to control)
Utah, Idaho (2001–2005) [45]	Rural Communities	Educational messages in examination rooms, pharmacies, newspapers, mailings. Key messages included “Do not treat viral infections with antibiotics”, how to manage respiratory tract infections, and ways to improve communication with the doctor	Computer decision support for treatment recommendations	Total Prescriptions /100 person-years	2.6 (95%CI[−3.7,9.4])	−8.8 (95% CI[−13.2,-4.2])	Not reported	Not reported
Massachusetts, Northwest Washington State (1996–1998) [27]	Managed Care Organization (MCO) Practices	CDC brochure mailings, waiting room materials	Meetings, CDC recommendations, feedback of prescribing rates	Children 3 to < 36 mo. old (antibiotics/person-year)	−11.5% (*P* < .0001)	−18.6% (*P* < .0001)	−16% (95%CI[− 8,-23%])^a^	< 0.01
				Children 36 to < 72 mo. old (antibiotics/person-year)	−9.7% (*P* < .0001)	−15% (*P* < .0001)	−12% (95%CI[− 2,-21%])^a^	< 0.01
Massachusetts (2000–2003) [28]	Community-wide	Materials consistent with CDC principles regarding antibiotic indications and resistance were distributed to offices, pharmacies, child care, and by mail	Kickoff dinners and educational materials, feedback about prescribing practices	Children 24 to < 48 months (antibiotics/person-year)	− 10.30%	−14.50%	−4.20%	< 0.01
				Children 48 to < 78 months (antibiotics/person-year)	−2.50%	−9.30%	−6.70%	< 0.0001
US Regions (2003–2005) [42]	Veterans Administration (VA) and non-VA emergency departments	CDC waiting room posters and brochures, interactive computer kiosk for information about viral infections	Educational sessions on judicious use, emphasizing CDC principles. Site-specific performance feedback	Antibiotics for Respiratory Tract Infections and Acute Bronchitis	0.5% (95%CI[− 3,5%])	−10% (95%CI[− 18,-2%])	Not reported	Not reported

^a^ Adjusted for baseline prescribing rates and other cofounders [27]

**Table 2:** Quasi Experimental Studies Demonstrating Decreased Antibiotic Prescription Rates in Response to Patient- or Public- Centered Educational Interventions Compared to Control

Location of Intervention (years observed)	Setting	Patient Education	Provider Education	Outcome Measure	Prescription Rate Change			
					Control	(Full) Intervention	Intervention Effect	*P* value (intervention effect compared to control)
Denver-Boulder Colorado (1996–1998) [32]	MCO practices	Household mailings and office-based educational materials regarding self care, when to expect antibiotics, and harmful effects of antibiotics	Education and meetings about management of acute bronchitis and how to say “no” to patients, site-specific prescribing rates	Antibiotics for Adults with Acute Bronchitis	−5% (*P* = 0.68)	−26% (*P* = 0.003)	Not reported	0.02
Denver, Colorado (2000–2001) [29]	MCO Practices	Household and office-based educational materials including CDC materials regarding resistance and facts about treatments for respiratory infections	Prescribing profiles and practice guidelines	Antibiotics for Adults with Acute Bronchitis	− 10% (local control), − 6% (distant control)	−24%	Not reported	0.006 (local control) < 0.002 (distant control)
Denver, Colorado (2002–2003) [30]	Community-wide	Media campaign with out-of-home advertising, office-based materials	Physician advocacy activities were mailed: postcards soliciting support, office materials, stethoscope clips	Antibiotic dispenses/1000 MCO members	Values not noted	−8.8% (*P* = 0.03)	Not reported	Not reported
Rural Alaska (1998–1999) [36]	Rural Communities	Villiage meetings, community fairs, high school classrooms, and news letters about respiratory infections and antibiotic resistance	Workshops for community health aids and physicians to review principles of appropriate use	Antibiotic Courses/person	−9.5% (*P* < 0.05)	−31% (*P* < 0.01)	Not reported	Not reported
Sacramento, California (1998–1999) [37]	MCO (clinic, urgent care)	Office-based materials and newsletter regarding indications for antibiotics, bacterial resistance, how to prevent infection, and how to take antibiotics	Clinical pharmacists presented CDC Judicious Use principles to physicians, nurse practitioners, and physician assistants. Provider-specific antibiotic prescribing profiles and cold kits were included.	Antibiotics for Acute Bronchitis	0%	−20% (*P* = 0.001)	Not reported	Not reported
Knox County, Tennessee (1997–1998) [43]	Community-wide	Printed materials and public media regarding indications for antibiotics	Lectures by a CDC physician and other presentations, prescribing guidelines, newsletters	Children < 15 years old (antibiotics/person-year)	−8%	−19%	− 11% (95%CI[− 8,-14%])	< 0.001
Utah (2001) [44]	Rural Community	Office-based informational brochures, media campaign about antibiotic resistance	Small group sessions overviewing antibiotic resistance and appropriate antibiotic use, algorithms	Upper respiratory tract infections treated with an antibiotic	−1.5% (*P* = 0.047)	− 15.6% (*P* = 0.002)	Not reported	0.006
Price, Rusk, Lincoln Counties, Wisconsin (1997) [24]	Community-wide	CDC pamphlets and posters distributed to clinics, pharmacies, child care facilities, schools	Grand rounds and small-group meetings regarding judicious use for pediatric respiratory infections, practice guidelines, CDC fact sheets	Solid antibiotic prescriptions/clinician	−8% (*P* = 0.934)	− 19% (*P* < 0.001)	− 11%	0.042
				Liquid antibiotic prescriptions/clinician	12% (*P* = 0.064)	−11% (*P* = 0.064)	−23%	0.019

Appropriateness of antibiotic prescribing was measured for three interventions. [22, 34, 45] Appropriateness was assessed by increases in first line therapy, [22, 34] use of antibiotics in the “never indicated” category, [45] and reductions in macrolide use. [34, 45] Significant improvements in were reported for all 3 of the interventions, though only one was a randomized-controlled trial. [45]

Knowledge, attitudes, and/or beliefs of the non-medical community were also commonly studied (10 interventions). Surveys were used to measure knowledge scores, expectations for treatment with antibiotics, or sense of communication efficacy. Statistically significant improvements in these measures were reported for 7 of these interventions (70%). When restricting to randomized controlled trials (5 of the 10 interventions), 60% showed statistically significant benefits. These studies frequently stratified on health insurance status and education level. Croft et al. found that the overall increase in knowledge was attributable to college-educated parents; there was no significant change in knowledge scores for non-college graduates. [25] In contrast, Trepka et al. found that higher education levels and private insurance, associated with higher baseline knowledge scores, was not associated with greater improvements in knowledge. [50] Bauchner et al., Huang et al., and Greene et al., found that participants who were less well educated or had lower baseline knowledge scores benefited the most from these interventions. [21, 33, 38] Huang et al. reasoned that parents of Medicaid-insured children, who started with lower baseline knowledge scores, may have limited access to health education and may therefore benefit the most from such interventions. [38]

Three interventions (10%) measured rates of penicillin non-susceptible *Streptococcus pneumoniae* pre- and post- intervention. [24, 36, 43] These studies were non-randomized, prospective controlled trials. None of these studies showed a lasting effect. Potential reasons provided for the lack of reductions in resistance included limited sample size, [43] short follow-up time, [24, 36, 43] and a need for greater reductions in the antibiotic prescribing rate than were evident for the population studied. [24]

Cost savings were estimated for two interventions (7%), [22, 30] attributed primarily to decreased prescription drug use. Gonzales et al. estimated a 3.1 ratio of health care savings to intervention costs. [30] However, no controlled analyses were performed.

### Interventions with provider education

While all interventions were required to have messages to the non-medical community to be included in our review, 19 of the 29 interventions (66%) additionally included an educational intervention for the medical community. These interventions are subanalyzed here.

#### Provider intervention characteristics

##### Target Audience

Every intervention that included messages for the medical community targeted physicians, with some interventions also specifying nurses (11%), physician assistants (16%), and nurse practitioners (21%).

##### Content of messages/mode of distribution

Of the 19 interventions targeting health professionals, 42% of included information about risks of inappropriate use. The vast majority of interventions employed tools for clinical decision making, such as algorithms, pathways, and guidelines for antibiotic prescribing (90%). Other commonly used methods were pathogen resistance surveillance data (42%) and audit/feedback specific for the physician or site (53%). CDC materials or principles of judicious use were frequently used (58%), like the non-medical community-only interventions. One intervention included financial incentives for physicians. [34]

##### Setting/mode of distribution

Most of the messages to medical personnel were distributed in the office or clinic setting (84%). The mode of distribution for these messages included group sessions or presentations, mailings, computer decision support systems, continuing medical education, and email.

#### Patient/public intervention characteristics

##### Target audience

All 19 interventions were directed to parents, though one also catered to children. Seven included information for Spanish speakers (37%).

##### Content

Sixteen of these interventions included information regarding appropriate use of antibiotics (84%). Twelve highlighted the risks of inappropriate use (63%). Lastly, 15 noted the use of CDC principles (79%).

##### Setting/Mode of Distribution

An average of 1.8 coded settings were utilized for distribution of these messages to the non-medical community. Most used the clinic or office setting (95%). Nine also distributed materials in community sites (47%). Seven interventions used direct mail (37%). Only one used the emergency department setting (5%). These interventions included an average of 1.9 coded modes of communication. These included print media (90%), mailed media (42%), public media (32%), presentations (26%), and videos (5%).

#### Outcomes

Antibiotic prescription rates were measured in all interventions that targeted both the medical and non-medical community. Most of these interventions reported decreased antibiotic use following the intervention (84%). Six of these interventions were randomized-controlled trials, five of which were effective (83%). Samore et al. compared a community-intervention alone to a community intervention plus clinical decision support; the community-only intervention had no significant impact on antibiotic prescribing rates, while the combined interventions led to a significant decline. [45]

Knowledge, attitudes, and/or beliefs of the lay community were analyzed in 3 of these interventions (16%), with 67% reporting a positive effect. However, only 1 of these was a randomized controlled trial; this trial was not effective.

Knowledge, attitude, and/or beliefs of the medical community were analyzed in 2 of these interventions, with one being effective (50%). However, the randomized controlled trial was not effective.

Appropriateness of physician antibiotic choice was analyzed in 3 of these interventions (16%), one of which was a randomized controlled trial. All were effective.

### Interventions without provider education

Of the 29 interventions, 10 were strictly public or patient directed. The results specific to these interventions are reported.

#### Intervention characteristics

##### Audience

All primarily targeted adults. Two interventions included materials for Spanish speakers (20%).

##### Content

Most included information about appropriate antibiotic use (80%) and risks of inappropriate use (90%). Five interventions noted use of CDC content (50%)

##### Setting/mode of distribution

An average of 1.2 coded settings were used. The majority used the clinic or office setting (60%). Other settings included community sites (30%), the emergency department (20%), or personal residence (10%). These interventions included an average of 1.8 coded modes of distribution, including print media (90%), video (40%), presentations (20%), public media (10%), SMS messaging (10%), and direct mail (10%).

#### Outcomes

Knowledge, attitudes, and/or beliefs of the non-medical community were most commonly studied (7 interventions). Of these, 5 were effective (71%). Four of these interventions were randomized controlled trials, 3 of which were effective (75%).

Prescription rates were measured for 3 of these interventions (30%). One of these was randomized controlled. None of these were effective at reducing prescription rates.

Adherence to prescription was measured for one of these interventions (10%). This was a randomized controlled trial that was not effective.

## Discussion

We found that most public messaging interventions focused on educating parents of young children through office-based posters and handouts, often produced by the Centers for Disease Control and Prevention. Common rationale for catering to this audience included the likely role of parental expectations in the high rates of inappropriate antibiotic use in children. [18, 20, 21, 26, 27, 38, 46, 50] Caregiver emotions may also affect adherence to practices such as watchful waiting aimed to decrease unnecessary antibiotic use. [52] Finkelstein et al. additionally explained that children may be at higher risk for spreading resistant organisms and are thus are an especially important population to target. [28]

Many interventions were multifaceted and distributed educational materials within the community as well as clinical sites. While we aimed to locate interventions targeting the public, over half of the interventions also included a clinician-education component. This was accomplished primarily through prescribing guidelines, audit and feedback of prescribing practices, and pathogen surveillance data, which were distributed through group presentations and mailings. The risks of antibiotic resistance were not as frequently communicated to the medical community, likely due to assumed prior knowledge of this target audience. The majority of interventions observed at least one favorable outcome, and improvements in antibiotic prescribing and patient knowledge, attitudes, and/or beliefs were reported at similar rates. When restricting our analysis to randomized controlled trials, the majority of interventions were still effective in their respective outcomes.

Thus, these interventions suggest that patient-directed messages can influence patient attitudes, knowledge, and beliefs about antibiotics, which are important factors in physician overprescribing. The success rate for this outcome was similar for interventions with or without a provider education component. Subanalyses included in some of the campaign evaluations suggested that educational interventions may be best directed to the Medicaid-insured and non-college graduates to ameliorate a lack of access to health education, although one study found the opposite. [25]

Most studies that measured antibiotic prescribing reported success. The addition of a clinician-directed component may be imperative for this reduction in antibiotic use. Every intervention that resulted in decreased antibiotic prescribing included a provider intervention. This contrasted with only 3 interventions measuring antibiotic prescribing that did not include a clinician component, none of which were effective. However, interventions that included clinician education were generally more comprehensive, as evidenced by the greater average number of settings and modes of distribution, which could explain that difference in rates of success. Regardless, clinician education may be an effective way to spread antibiotic awareness among the community indirectly. One international intervention evaluated provider communication training and found it to be effective in lowering antibiotic prescribing for both upper and lower respiratory tract infections. [53] In this study, physicians were provided internet-based training on patient-centered communication. Thus, educating providers on how to educate patients may be a worthwhile strategy for future antibiotic stewardship campaigns.

It is unclear whether these observed changes in antibiotic prescribing are influencing antibiotic resistance rates. One study measured short-term decreases in *Streptococcal pneumoniae* rates, but these changes were not sustained. [36] Longitudinal studies are likely required to verify changes in susceptibility patterns in response to lay person-centered educational interventions.

The measured changes in prescription drug use have the potential to streamline the spending of limited healthcare funds. However, costs were not commonly studied in the analyses of these interventions, representing a general gap of knowledge that could motivate future implementation of similar programs.

## Limitations

This systematic review has several strengths, including a rigorous search strategy developed with the assistance of a reference librarian and a highly reliable classification scheme. However, our review has several limitations. It is possible that relevant interventions were missed, though the assistance of a trained librarian in the formation of our search strategy, as well as our secondary search of all references, likely reduced this probability. Secondly, meta-analysis of intervention effectiveness was not performed due to heterogeneity among the interventions in terms of audience, messages, delivery, and outcomes. Additionally, some retrieved articles did not describe the content of the educational materials in much detail; various interventions likely included messages about appropriate antibiotic use and risks of antibiotic overuse that were not included in the calculations. Thus, the descriptive statistics for these measures likely represent an underestimate. Further, the effectiveness of educational interventions reported here is dependent on the quality of the included articles. For example, some quasi experimental studies may have poorly-matched comparison groups, though we hoped to ameliorate this by reporting descriptive statistics for the subset of randomized-controlled trials. Publication bias could also lead to the overestimation of educational intervention effectiveness.

## Conclusion

With increasing antimicrobial resistance, stimulated in part by overuse of antibiotics in healthcare settings, changing the beliefs and actions of both the lay and the medical communities is important. Previous educational programs and public messaging campaigns have resulted in substantial declines in antibiotic use and should be used as models for succeeding efforts to curb inappropriate antibiotic use and subsequent antibiotic resistance. Future interventions should consider both the lay and medical communities, and target appropriate use as well as potential adverse effects of antibiotics. Furthermore, a multifaceted approach may be most effective in changing patient perspectives and reducing antibiotic prescribing. Resources from the Centers for Disease Control and Prevention appear to be an effective and easily-implemented way to inform the lay community. Among the most effective campaigns, clinician decision-making tools and prescribing profiles are commonly employed. These methods have demonstrated the ability to reduce antibiotic prescribing both within individual healthcare organizations as well as community wide.

While existing research is limited, our systematic review provides evidence that patient-directed messaging is sufficient to change antibiotic-related knowledge, attitudes, and/or beliefs. It has not yet been shown whether these changes in perspectives can affect antibiotic prescribing in the absence of medical provider intervention. Given that the most impactful campaigns have included a clinician outreach component, more research is needed to determine how we can best integrate the public awareness and clinician activation strategies in future campaigns. In addition, further research is also needed to evaluate whether the observed changes in antibiotic prescribing are sufficient to impact antibiotic susceptibility long-term.

## Additional file

Additional file 1: **Appendices A-D**. Appendix A delineates the individualized search strategies for PubMed, Google Scholar, Embase, CINAHL, and Scopus. Appendix B includes article localization and article study design. Appendix C lists the antibiotic educational interventions and their associated articles. Appendix D is a table of coded intervention characteristics. (DOCX 141 kb)

## Data Availability

All data generated or analyzed during this study are included in this published article and its supplementary information files.

## Abbreviations

CDC: Centers for Disease Control and Prevention
CME: Continuing Medical Education
KAB: Knowledge, attitude, and/or beliefs
SMS: Short Message Service
