# Supplementary material for: Communication interventions to promote the public’s awareness of antibiotics: a systematic review
Source: BMC Public Health. 2019 Jul 8;19:899. doi: 10.1186/s12889-019-7258-3 (PMC6615171; doi:10.1186/s12889-019-7258-3)
Supplement: Supplementary file 1 — Appendices A-D. Appendix A delineates the individualized search strategies for PubMed, Google Scholar, Embase, CINAHL, and Scopus. Appendix B includes article localization and article study design. Appendix C lists the antibiotic educational interventions and their associated articles. Appendix D is a table of coded intervention characteristics. (DOCX 141 kb) [file 12889_2019_7258_MOESM1_ESM.docx]

**Appendix A.**

PUBMED:

1. Subject: Antibiotics

("Anti-Bacterial Agents"[Mesh] OR antibiotic OR antibiotics OR "antimicrobial" OR "antimicrobials")

1. Mode of Intervention: Messaging/campaign

“Mass media”[Mesh] OR “Communications media”[Mesh] OR “Audiovisual Aids”[Mesh] OR “Text Messaging”[Mesh] OR “Government Programs”[Mesh] OR “National Health Programs”[Mesh] OR “public health/methods”[Mesh] OR “media campaign” OR “media campaigns” OR “public health campaigns” OR “public campaign” OR “public campaigns” OR “community-wide campaign” OR “community campaign” OR “message program” OR “messaging program” OR “messaging campaign” OR “educational messages” OR “community intervention” OR “community-level intervention” OR “social media” OR “communication campaign"

1. Outcome: Public Awareness

“Health promotion”[Mesh] OR “health education”[Mesh] OR “Patient Education as Topic/methods”[Mesh] OR “Parents/education”[Mesh] OR "Health Knowledge, attitudes, Practice"[Mesh] OR "Patient Acceptance of Health Care"[Mesh] OR “Attitude to Health”[Mesh] OR awareness

Search Input 02/05/16:

1. Subject: Antibiotics (789548)
2. Intervention: Messaging/Campaign (596895)
3. Outcome: Public Awareness (562499)
4. 1 and 2 and 3 (567)
5. 4 and United States (120)
6. 5 and English language filter (120)
7. 6 and Human species filter (119)

GOOGLE SCHOLAR:

Search Input 02/16/16:

Filters: English language, Years 1996-2016

antibiotic|antibiotics|antimicrobial|antimicrobials "public health campaign"|"public campaign"|"messaging campaign"|”media campaign”|"community campaign"|"mass media campaign" “public awareness”|”patient education” "United States”

482 Results

EMBASE:

1. Subject: Antibiotics

'antibiotic*':ab,ti OR antimicrobial*:ab,ti

1. Mode of Intervention: Messaging/campaign

'mass media'/exp OR 'mass media' OR 'public health campaign*' OR 'public health campaign'/exp OR 'public health campaign' OR 'media campaign*' OR 'public campaign*' OR 'community campaign*' OR 'community-wide campaign*' OR 'messaging program*' OR 'message program*' OR 'messaging campaign*' OR 'educational message*' OR 'community intervention*' OR 'community-level intervention' OR 'community based' OR 'social media'/exp OR 'social media' OR 'communication campaign*'

1. Outcome: Public Awareness

'health promotion'/exp OR 'health promotion' OR 'health education'/exp OR 'health education' OR 'patient education as topic'/exp OR 'patient education as topic' OR 'parents education' OR 'health knowledge attitudes practice'/exp OR 'health knowledge attitudes practice' OR 'patient acceptance of health care'/exp OR 'patient acceptance of health care' OR 'attitude to health'/exp OR 'attitude to health' OR 'awareness'/exp OR 'awareness'

1. Location: United States

'united states'/exp OR 'united states' OR 'usa'/exp OR 'usa' OR 'us'

Search Input 02/16/16:

1. Subject: Antibiotics (430,885)
2. Intervention: Messaging/Campaign (79,850)
3. Outcome: Public Awareness (748,744)
4. Location United States (13,838,924)
5. 1 AND 2 AND 3 AND 4 (66)
6. 5 AND [article]/lim AND [english]/lim AND [humans]/lim (43)

CINAHL:

1. Subject: Antibiotics

(MM "Antibiotics") OR "antibiotics" OR (MM "Drug Resistance, Microbial")

1. Intervention: Messaging/Campaign

(MM "Communications Media") OR (MM "Social Media") OR “mass media” OR “communications media” OR “text messaging” OR “government programs” OR “national health programs” OR “public health” OR “media campaign” OR “media campaigns” OR “public health campaigns” OR “community-wide campaign” OR “community campaign” OR “message program” OR “messaging program” OR “messaging campaign” OR “educational messages” OR “community intervention” OR “community-level intervention” OR “social media” OR “communication campaign”

1. Outcome: Public Awareness

(MH "Attitude to Health+") OR (MH "Attitude of Health Personnel+") OR (MM "Health Promotion") OR (MM "Health Education") OR “Health promotion” OR “health education” OR “Patient Education” OR “Parents education” OR awareness

Search Input 02/16/16:

1. Subject: Antibiotics (45,490)
2. Intervention: Messaging/Campaign (133,822)
3. Outcome: Public Awareness (309,275)
4. 1 AND 2 AND 3 (112)
5. 4 AND Limiters: English Language, Research Article (59)
6. 5 AND Geography: USA (23)

SCOPUS:

1. Subject: Antibiotics (Title)

antibiotic OR antibiotic OR antimicrobial OR antimicrobials

1. Intervention: Messaging/Campaign

“mass media” OR “communications media” OR “text messaging” OR “government programs” OR “national health programs” OR “public health” OR “media campaign” OR “media campaigns” OR “public health campaigns” OR “community-wide campaign” OR “community campaign” OR “message program” OR “messaging program” OR “messaging campaign” OR “educational messages” OR “community intervention” OR “community-level intervention” OR “social media” OR “communication campaign”

1. Outcome: Public Awareness

“Health promotion” OR “health education” OR “Patient Education” OR “Parents education” OR awareness

1. Location: United States

“United States” OR US OR USA

Search Input 02/05/16:

1. Subject: Antibiotics (756,965)
2. Intervention: Messaging/Campaign (423,972)
3. Outcome: Public Awareness (534,303)
4. Location: United States (1,636,440)
5. 1 AND 2 AND 3 AND 4 (131)
6. 5 AND Filter: Article (95)
7. 6 AND Filter: United States (47)

**Appendix B.**

| **Appendix B. Article Localization and Study Design** | | |
| --- | --- | --- |
| **Article (n = 34)** | **Identification Method** | **Study Design** |
| Alder et al. (2005) [^18^](#_ENREF_18) | Secondary Search | Randomized Controlled Trial |
| Alder et al. (2010) [^19^](#_ENREF_19) | CINAHL | Multi-Level Logistical Regression |
| Ashe et al. (2006) [^20^](#_ENREF_20) | PubMed | Prospective, Non-randomized: Pre/post study |
| Bauchner et al. (2001) [^21^](#_ENREF_21) | Secondary Search | Randomized Controlled Trial |
| Bell (2002) [^22^](#_ENREF_22) | PubMed | Descriptive Study |
| Belongia et al. (2001) [^24^](#_ENREF_24) | PubMed | Prospective, Non-randomized: Control population |
| Belongia et al. (2005) [^23^](#_ENREF_23) | PubMed | Prospective, Non-randomized: Control population |
| Croft et al. (2007) [^25^](#_ENREF_25) | Secondary Search | Randomized Controlled Trial: Group-randomized |
| Doyne et al. (2004) [^26^](#_ENREF_26) | Secondary Search | Randomized Controlled Trial: Group-randomized |
| Finkelstein et al. (2001) [^27^](#_ENREF_27) | Secondary Search | Randomized Controlled Trial: Group-randomized |
| Finkelstein et al. (2008) [^28^](#_ENREF_28) | PubMed | Randomized Controlled Trial: Cluster-randomized |
| Gonzales et al. (1999) [^32^](#_ENREF_32) | Secondary Search | Prospective, Non-randomized: Control population |
| Gonzales et al. (2004) [^31^](#_ENREF_31) | Secondary Search | Prospective, Non-randomized: Control population |
| Gonzales et al. (2005) [^29^](#_ENREF_29) | Secondary Search | Prospective, Non-randomized: Control population |
| Gonzales et al. (2008) [^30^](#_ENREF_30) | PubMed | Prospective, Non-randomized: Control population |
| Greene et al. (2004) [^34^](#_ENREF_34) | Embase | Prospective, Non-randomized: Pre/post study |
| Greene et al. (2011) [^33^](#_ENREF_33) | Google Scholar | Prospective, Non-randomized: Pre/post study |
| Harris et al. (2003) [^35^](#_ENREF_35) | Secondary Search | Prospective, Non-randomized: Pre/post study |
| Hennessy et al. (2002) [^36^](#_ENREF_36) | Secondary Search | Prospective, Non-randomized: Control population |
| Hickman et al. (2003) [^37^](#_ENREF_37) | Embase | Prospective, Non-randomized: Control population |
| Huang et al. (2007) [^38^](#_ENREF_38) | Secondary Search | Randomized Controlled Trial: Cluster-randomized |
| Kiang et al. (2005) [^39^](#_ENREF_39) | PubMed | Prospective, Non-randomized: Control population |
| Mainous et al. (2000) [^41^](#_ENREF_41) | Secondary Search | Randomized Controlled Trial |
| Mainous et al. (2009) [^40^](#_ENREF_40) | PubMed | Prospective, Non-randomized: Control population |
| Metlay et al. (2007) [^42^](#_ENREF_42) | Secondary Search | Randomized Controlled Trial: Cluster-randomized |
| Perz et al. (2002) [^43^](#_ENREF_43) | PubMed | Prospective, Non-randomized: Control population |
| Rubin et al. (2005) [^44^](#_ENREF_44) | PubMed | Prospective, Non-randomized: Comparison population |
| Samore et al. (2005) [^45^](#_ENREF_45) | Secondary Search | Randomized Controlled Trial: Cluster-randomized |
| Schnellinger et al. (2010) [^46^](#_ENREF_46) | PubMed | Randomized Controlled Trial |
| Stille et al. (2008) [^47^](#_ENREF_47) | PubMed | Randomized Controlled Trial: Cluster-randomized |
| Suffoletto et al. (2012) [^48^](#_ENREF_48) | Embase | Randomized Controlled Trial |
| Taylor et al. (2005) [^49^](#_ENREF_49) | Secondary Search | Randomized Controlled Trial |
| Trepka et al. (2001) [^50^](#_ENREF_50) | PubMed | Prospective, Non-randomized: Control population |
| Wheeler et al. (2001) [^51^](#_ENREF_51) | PubMed | Prospective, Non-randomized: Control population |

**Appendix C.**

| **Appendix C. Antibiotic Education Interventions and Associated Articles** | |
| --- | --- |
| **Intervention Location (years observed) (n=29)** | **Associated Campaigns/Organizations** |
| Salt Lake City, Utah (2000) [^18^](#_ENREF_18) | Using Antibiotics Wisely |
| Utah, Idaho (2001-2005) [^19^](#_ENREF_19)^,^[^45^](#_ENREF_45) | Inter-Mountain Project on Antimicrobial Resistance and Therapy (IMPART) |
| Westchester County, New York (2001) [^20^](#_ENREF_20) |  |
| Boston, Massachusetts [^21^](#_ENREF_21) |  |
| Iowa (1998-1999) [^22^](#_ENREF_22) | Iowa Department of Health Task Force |
| Wisconsin (2000-2003) [^23^](#_ENREF_23)^,^[^39^](#_ENREF_39) | Wisconsin Antibiotic Resistance Network (WARN) |
| Wisconsin (2001) [^25^](#_ENREF_25) | Wisconsin Antibiotic Resistance Network (WARN) |
| Southwest Ohio, Northern Kentucky (2001-2002) [^26^](#_ENREF_26) |  |
| Massachusetts, Northwest Washington State (1996-1998) [^27^](#_ENREF_27) |  |
| Denver-Boulder Colorado (1996-1998) [^32^](#_ENREF_32) |  |
| Denver, Colorado (2001-2002) [^31^](#_ENREF_31) |  |
| Denver, Colorado (2000-2001) [^29^](#_ENREF_29) | Minimizing Antibiotic Resistance in Colorado Project (pilot project for Get Smart Colorado) |
| Denver, Colorado (2002-2003) [^30^](#_ENREF_30) | Get Smart Colorado |
| 9 counties surrounding Rochester, NY (2000-2001) [^34^](#_ENREF_34) |  |
| Charlotte, North Carolina (2008) [^33^](#_ENREF_33) |  |
| Denver, Colorado (2000-2001) [^35^](#_ENREF_35) |  |
| Rural Alaska (1999-2000) [^36^](#_ENREF_36) |  |
| Sacramento, California (1998-1999) [^37^](#_ENREF_37) |  |
| Massachusetts (2000-2003) [^28^](#_ENREF_28)^,^[^38^](#_ENREF_38)^,^[^47^](#_ENREF_47) | Reducing Antibiotics for Children in Massachusetts (REACH) |
| Kentucky (1996-1997) [^41^](#_ENREF_41) |  |
| Charleston, South Carolina (2007-2008) [^40^](#_ENREF_40) | Solo Con Receta |
| US Regions (2003-2005) [^42^](#_ENREF_42) | Improving Antibiotic Use in Acute Care Treatment (IMPAACT) |
| Knox County, Tennessee (1997-1998) [^43^](#_ENREF_43) |  |
| Utah (2001) [^44^](#_ENREF_44) |  |
| Minnesota (2008-2009) [^46^](#_ENREF_46) |  |
| Western Pennsylvania (2011) [^48^](#_ENREF_48) |  |
| Seattle, Washington (2000-2002) [^49^](#_ENREF_49) |  |
| Price, Rusk, Lincoln Counties, Wisconsin (1997) [^24^](#_ENREF_24)^,^[^50^](#_ENREF_50) | Marshfield Medical Research Foundation |
| Central Arkansas (1999-2000) [^51^](#_ENREF_51) |  |

**Appendix D.**

| **Appendix D. Intervention Characteristics** | | | | | | | | | | | | | | | | | | | | | | | | | | | | | | | |
| --- | --- | --- | --- | --- | --- | --- | --- | --- | --- | --- | --- | --- | --- | --- | --- | --- | --- | --- | --- | --- | --- | --- | --- | --- | --- | --- | --- | --- | --- | --- | --- |
|  | **Intervention** | | | | | | | | | | | | | | | | | | | | | | | | | | | | | | |
|  | **1** | **2** | **3** | **4** | **5** | **6** | **7** | **8** | **9** | **10** | **11** | **12** | **13** | **14** | **15** | **16** | **17** | **18** | **19** | **20** | **21** | **22** | **23** | **24** | **25** | **26** | **27** | **28** | **29** | **Total** | **% (n=29)** |
|  | [**^18^**](#_ENREF_18) | [**^19^**](#_ENREF_19)**^,^**[**^45^**](#_ENREF_45) | [**^20^**](#_ENREF_20) | [**^21^**](#_ENREF_21) | [**^22^**](#_ENREF_22) | [**^23^**](#_ENREF_23)**^,^**[**^39^**](#_ENREF_39) | [**^25^**](#_ENREF_25) | [**^26^**](#_ENREF_26) | [**^27^**](#_ENREF_27) | [**^32^**](#_ENREF_32) | [**^31^**](#_ENREF_31) | [**^29^**](#_ENREF_29) | [**^30^**](#_ENREF_30) | [**^34^**](#_ENREF_34) | [**^33^**](#_ENREF_33) | [**^35^**](#_ENREF_35) | [**^36^**](#_ENREF_36) | [**^37^**](#_ENREF_37) | [**^28^**](#_ENREF_28)**^,^**[**^38^**](#_ENREF_38)**^,^**[**^47^**](#_ENREF_47) | [**^41^**](#_ENREF_41) | [**^40^**](#_ENREF_40) | [**^42^**](#_ENREF_42) | [**^43^**](#_ENREF_43) | [**^44^**](#_ENREF_44) | [**^46^**](#_ENREF_46) | [**^48^**](#_ENREF_48) | [**^49^**](#_ENREF_49) | [**^24^**](#_ENREF_24)**^,^**[**^50^**](#_ENREF_50) | [**^51^**](#_ENREF_51) |  |  |
| **Direct Target Audience** |  |  |  |  |  |  |  |  |  |  |  |  |  |  |  |  |  |  |  |  |  |  |  |  |  |  |  |  |  |  |  |
| Adult | X | X | X | X | X | X | X | X | X | X | X | X | X | X | X | X | X | X | X | X | X | X | X | X | X | X | X | X | X | 29 | 100 |
| "Parents" Specified | X | X | X | X |  | X | X | X | X |  |  |  | X |  |  |  |  | X | X | X |  |  | X | X | X |  | X | X | X | 18 | 62.1 |
| Children/Student |  |  |  |  |  |  |  |  |  |  |  |  |  |  |  |  | X |  |  |  |  |  |  |  |  |  |  |  |  | 1 | 3.4 |
| Spanish Materials Included |  | X |  |  |  | X |  |  |  |  | X | X | X |  | X | X |  |  |  |  | X | X |  |  |  |  |  |  |  | 9 | 31 |
| **Content of Message** |  |  |  |  |  |  |  |  |  |  |  |  |  |  |  |  |  |  |  |  |  |  |  |  |  |  |  |  |  |  |  |
| Appropriate Use | X | X | X | X | X | X | X | X |  | X | X | X | X | X | X | X | X | X | X |  |  | X | X |  | X |  | X | X | X | 24 | 82.8 |
| Risks of Inappropriate Use | X | X | X | X | X |  | X | X |  | X | X | X | X |  | X |  | X | X | X |  | X | X |  | X | X |  | X |  | X | 21 | 72.4 |
| Noted Use of CDC Materials/ Principles | X | X | X |  | X | X |  | X | X | X | X |  | X |  | X |  | X | X | X | X |  | X | X |  |  |  | X | X | X | 20 | 69 |
| Other | X |  |  | X |  |  | X | X |  | X |  | X | X |  | X | X |  | X |  |  |  |  |  |  |  | X |  |  | X | 12 | 41.4 |
| **Point of Message Distribution** |  |  |  |  |  |  |  |  |  |  |  |  |  |  |  |  |  |  |  |  |  |  |  |  |  |  |  |  |  |  |  |
| Emergency Department |  |  |  |  |  |  |  |  |  |  |  |  |  |  |  |  |  |  |  |  |  | X |  |  | X | X |  |  |  | 3 | 10.3 |
| Office/Clinic (including urgent care) | X | X | X | X | X | X |  | X | X | X | X | X | X | X |  | X | X | X | X | X | X |  | X | X |  |  | X | X | X | 24 | 82.8 |
| Community Site |  | X |  |  | X | X | X |  |  |  |  |  | X |  | X |  | X |  | X |  | X |  | X | X |  |  |  | X |  | 12 | 41.4 |
| Child Care Center |  |  |  |  |  | X | X |  |  |  |  |  |  |  |  |  |  |  | X |  |  |  | X |  |  |  |  | X |  | 5 | 17.2 |
| Local Pharmacy |  | X |  |  |  | X |  |  |  |  |  |  |  |  | X |  |  |  | X |  |  |  | X |  |  |  |  | X |  | 6 | 20.7 |
| Community Fair |  | X |  |  |  | X |  |  |  |  |  |  | X |  |  |  | X |  |  |  |  |  |  |  |  |  |  |  |  | 4 | 13.8 |
| Restaurants/Food Stores |  |  |  |  |  |  |  |  |  |  |  |  |  |  |  |  |  |  |  |  | X |  |  |  |  |  |  |  |  | 1 | 3.4 |
| Church |  | X |  |  |  |  |  |  |  |  |  |  |  |  |  |  |  |  |  |  | X |  |  |  |  |  |  |  |  | 2 | 6.9 |
| School |  | X |  |  |  |  |  |  |  |  |  |  |  |  |  |  | X |  |  |  | X |  |  |  |  |  |  | X |  | 4 | 13.8 |
| Personal Residence-Public Media |  | X |  |  | X | X |  |  |  |  |  |  | X |  |  |  |  |  |  |  | X |  | X | X |  |  |  |  |  | 7 | 24.1 |
| Personal Residence-Direct Mail |  | X |  |  |  |  |  |  | X | X | X | X |  |  |  |  | X |  | X |  |  |  |  |  |  |  | X |  |  | 8 | 27.6 |
| Other |  | X |  |  | X |  |  |  |  |  |  |  |  |  |  |  | X |  |  |  |  |  |  |  |  |  |  | X |  | 4 | 13.8 |
| **Channel/Mode of Communication** |  |  |  |  |  |  |  |  |  |  |  |  |  |  |  |  |  |  |  |  |  |  |  |  |  |  |  |  |  |  |  |
| Print Media | X | X | X | X | X | X | X | X | X | X | X | X | X | X | X |  |  | X | X | X | X | X | X | X | X |  | X | X | X | 26 | 89.7 |
| Handouts | X | X |  | X | X | X | X | X | X | X | X | X | X | X | X |  |  | X | X | X | X | X | X | X | X |  | X | X | X | 25 | 86.2 |
| Posters |  | X | X |  | X | X | X | X | X | X | X | X | X |  | X |  |  | X | X |  |  | X |  |  |  |  |  | X |  | 16 | 55.2 |
| Mailed Media |  | X |  |  |  | X |  |  | X | X | X | X |  |  |  |  | X |  | X |  |  |  |  |  |  |  | X |  |  | 9 | 31 |
| Public Media |  | X |  |  | X | X |  |  |  |  |  |  | X |  |  |  |  |  |  |  | X |  | X | X |  |  |  |  |  | 7 | 24.1 |
| Radio |  |  |  |  | X | X |  |  |  |  |  |  | X |  |  |  |  |  |  |  | X |  | X |  |  |  |  |  |  | 5 | 17.2 |
| Television |  |  |  |  |  | X |  |  |  |  |  |  | X |  |  |  |  |  |  |  |  |  | X |  |  |  |  |  |  | 3 | 10.3 |
| Newspaper |  | X |  |  | X | X |  |  |  |  |  |  | X |  |  |  |  |  |  |  | X |  | X |  |  |  |  |  |  | 6 | 20.7 |
| Outdoor Advertising |  |  |  |  |  |  |  |  |  |  |  |  | X |  |  |  |  |  |  |  |  |  |  |  |  |  |  |  |  | 1 | 3.4 |
| Video Messaging |  |  |  | X |  |  |  |  |  |  |  |  |  |  |  |  |  |  |  |  |  | X |  |  | X |  | X |  | X | 5 | 17.2 |
| SMS Messaging |  |  |  |  |  |  |  |  |  |  |  |  |  |  |  |  |  |  |  |  |  |  |  |  |  | X |  |  |  | 1 | 3.4 |
| Presentations |  | X |  |  |  | X | X |  |  |  |  |  | X |  | X |  | X |  |  |  |  |  |  |  |  |  |  | X |  | 7 | 24.1 |
| Other | X |  |  |  | X | X |  |  |  |  |  |  | X |  |  | X | X |  |  |  |  |  | X |  |  |  |  |  |  | 7 | 24.1 |
| **Medical Community Intervention Included** |  | X |  |  | X | X |  | X | X | X | X | X | X | X |  | X | X | X | X | X |  | X | X | X |  |  |  | X |  | 19 | 65.5 |
| **Direct Target Audience** |  |  |  |  |  |  |  |  |  |  |  |  |  |  |  |  |  |  |  |  |  |  |  |  |  |  |  |  |  |  |  |
| Physicians |  | X |  |  | X | X |  | X | X | X | X | X | X | X |  | X | X | X | X | X |  | X | X | X |  |  |  | X |  | 19 |  |
| Nurses |  |  |  |  | X |  |  |  |  | X |  |  |  |  |  |  |  |  |  |  |  |  |  |  |  |  |  |  |  | 2 |  |
| Physician Assistants |  |  |  |  | X |  |  |  |  | X |  |  |  |  |  |  |  | X |  |  |  |  |  |  |  |  |  |  |  | 3 |  |
| Nurse Practitioners |  |  |  |  | X |  |  |  |  | X |  |  |  |  |  | X |  | X |  |  |  |  |  |  |  |  |  |  |  | 4 |  |
| **Content of Messages** |  |  |  |  |  |  |  |  |  |  |  |  |  |  |  |  |  |  |  |  |  |  |  |  |  |  |  |  |  |  |  |
| Risks of Inappropriate Use |  |  |  |  | X |  |  |  | X | X |  |  |  |  |  | X |  | X | X |  |  |  |  | X |  |  |  | X |  | 8 |  |
| Pathogen Resistance Surveillance Data |  |  |  |  | X | X |  | X |  | X |  |  |  |  |  | X |  | X | X |  |  |  |  | X |  |  |  |  |  | 8 |  |
| Algorithms/ Pathways/ Guidelines |  | X |  |  | X | X |  | X | X | X | X | X | X | X |  | X |  | X | X |  |  | X | X | X |  |  |  | X |  | 17 |  |
| Audit/Feedback for physician or site |  |  |  |  | X |  |  | X | X | X | X | X |  | X |  |  |  |  | X | X |  | X |  |  |  |  |  |  |  | 10 |  |
| Noted Use of CDC materials/ principals |  |  |  |  | X |  |  |  | X |  |  | X | X |  |  | X | X | X | X |  |  | X | X |  |  |  |  | X |  | 11 |  |
| Cold Kits |  |  |  |  |  |  |  |  |  |  |  |  |  |  |  |  |  | X |  |  |  |  |  |  |  |  |  | X |  | 2 |  |
| Other |  |  |  |  |  |  |  | X |  | X |  | X |  | X |  |  |  | X | X |  |  |  |  |  |  |  |  |  |  | 6 |  |
| **Location of Received Message** |  |  |  |  |  |  |  |  |  |  |  |  |  |  |  |  |  |  |  |  |  |  |  |  |  |  |  |  |  |  |  |
| Emergency Department |  |  |  |  |  |  |  |  |  |  |  |  |  |  |  |  |  |  |  |  |  | X |  |  |  |  |  |  |  | 1 |  |
| Office/Clinic |  | X |  |  | X | X |  | X | X | X | X | X | X | X |  | X |  | X | X |  |  |  | X | X |  |  |  | X |  | 16 |  |
| Personal Residence |  |  |  |  |  |  |  |  |  |  |  |  |  |  |  |  |  |  |  |  |  |  |  |  |  |  |  |  |  | 0 |  |
| Other |  |  |  |  |  |  |  |  |  |  |  |  |  | X |  |  |  |  | X |  |  |  |  |  |  |  |  |  |  | 2 |  |
| **Channel/Mode of Communication** |  |  |  |  |  |  |  |  |  |  |  |  |  |  |  |  |  |  |  |  |  |  |  |  |  |  |  |  |  |  |  |
| Computer Decision Support |  | X |  |  |  |  |  |  |  |  |  |  |  |  |  |  |  |  |  |  |  |  |  |  |  |  |  |  |  | 1 |  |
| Group Sessions/ Presentations |  | X |  |  |  | X |  | X | X | X |  |  |  | X |  | X | X | X | X |  |  | X |  | X |  |  |  | X |  | 13 |  |
| Email |  |  |  |  |  |  |  |  |  |  |  |  |  |  |  |  |  |  | X |  |  |  |  |  |  |  |  |  |  | 1 |  |
| Mailings |  |  |  |  | X | X |  | X | X |  |  | X | X | X |  |  |  |  | X |  |  |  |  |  |  |  |  |  |  | 8 |  |
| CME |  | X |  |  |  | X |  |  |  |  |  |  |  |  |  |  |  |  |  |  |  |  | X |  |  |  |  |  |  | 3 |  |
| Other |  |  |  |  |  | X |  |  |  |  | X | X | X |  |  | X |  |  |  |  |  |  | X |  |  |  |  |  |  | 6 |  |
| **Major Outcomes (Effective = Y, Not Effective = N)** |  |  |  |  |  |  |  |  |  |  |  |  |  |  |  |  |  |  |  |  |  |  |  |  |  |  |  |  |  |  |  |
| Knowledge/ Attitude/ Beliefs: Medical Community |  |  |  |  |  | Y |  |  |  |  |  |  |  |  |  |  |  |  | N |  |  |  |  |  |  |  |  |  |  | 2 | 6.9 |
| Knowledge/ Attitude/ Beliefs: Non-Medical Community | Y | Y |  | N |  |  | Y |  |  |  |  |  |  |  | Y |  |  |  | N |  | N |  |  |  | Y |  |  | Y | Y | 10 | 34.5 |
| Antibiotic Prescription Rates |  | Y | N |  | Y | N |  | N | Y | Y | N | Y | Y | Y |  | Y | Y | Y | Y | Y |  | Y | Y | Y |  |  | N | Y | N | 22 | 75.9 |
| Appropriateness of Antibiotic Use |  | Y |  |  | Y |  |  |  |  |  |  |  |  | Y |  |  |  |  |  |  |  |  |  |  |  |  |  |  |  | 3 | 10.3 |
| Adherence to Prescription |  |  |  |  |  |  |  |  |  |  |  |  |  |  |  |  |  |  |  |  |  |  |  |  |  | N |  |  |  | 1 | 3.4 |
| Resistance Patterns |  |  |  |  |  |  |  |  |  |  |  |  |  |  |  |  | N |  |  |  |  |  | N |  |  |  |  | N |  | 3 | 10.3 |
| Cost Savings |  |  |  |  | Y |  |  |  |  |  |  |  | Y |  |  |  |  |  |  |  |  |  |  |  |  |  |  |  |  | 2 | 6.9 |
| Reduced Healthcare Visits |  |  |  |  |  |  |  |  |  |  |  |  | Y |  |  |  | Y |  |  |  |  |  | N |  |  |  |  |  |  | 3 | 10.3 |
| Campaign Exposure (compared to control population) |  |  |  |  |  |  |  |  |  |  |  |  | Y |  |  |  |  |  |  |  |  |  |  |  |  |  |  | Y |  | 2 | 6.9 |
